# Cancer Immunotherapy: The Role of Nursing in Patient Education, Assessment, Monitoring, and Support

**DOI:** 10.3390/curroncol32070392

**Published:** 2025-07-09

**Authors:** Parmis Mirzadeh, Edith Pituskin, Ivan Au, Sheri Sneath, Catriona J. Buick

**Affiliations:** 1 Faculty of Health, York University, Toronto, ON M3J 1P3, Canada; mirzadeh@yorku.ca; 2 Faculty of Nursing, University of Alberta, Edmonton, AB T6G 2R3, Canada; pituskin@ualberta.ca; 3 Alberta Health Services, Cancer Care, Edmonton, AB T2S 3C3, Canadasheri.sneath@albertahealthservices.ca (S.S.); 4 Sunnybrook Research Institute, Toronto, ON M4N 3M5, USA

**Keywords:** cancer immunotherapy, immune checkpoint inhibitors, nursing

## Abstract

The prevalence of cancer is rising both in Canada and across the world, with approximately 35 million new cases predicted by 2050. Cancer immunotherapy is a form of treatment that harnesses the body’s immune system to fight cancer cells, increasing life expectancy beyond what traditional treatments offer. Immunotherapy may cause immune-related adverse events that differ from the toxicities of traditional treatments. While these events can be detrimental to health, it is critical that they are caught early. This perspective paper examines the evolving role of oncology nurses within the cancer care continuum in caring for patients receiving cancer immunotherapy, specifically immune checkpoint inhibitors. Oncology nurses provide care in many areas, specifically in educating patients on the early detection of side effects to prevent negative outcomes, assessing and monitoring patient symptoms through a variety of means, including nurse-led clinics, and providing support to patients undergoing therapy. This work helps identify gaps in the literature. Future research is required for advancing cancer immunotherapies and better detecting early signs of side effects for nurses practicing in different settings, ensuring timely care.

## Simple Summary

Nearly half of North American adults are expected to have cancer at some point in their lives. “New” treatments like immunotherapy show great promise in helping people live significantly longer by using the body’s own defenses to target cancer. While immunotherapy is an effective form of cancer treatment, it can cause unique side effects, presenting a new challenge for both patients and healthcare providers. To prevent the escalation of side effects into negative health outcomes, it is important to educate patients on detecting side effects early, assessing and monitoring their symptoms, and providing support. Oncology nurses have a central role and responsibility in this process; therefore, this paper focuses on the evolving role of nurses in the cancer care continuum for patients receiving immunotherapy. More research is needed to enhance cancer immunotherapy treatments and better understand how to detect side effects earlier.

## 1. Introduction

The prevalence of cancer is rising both in Canada and across the world, with approximately 35 million total new cases predicted by the year 2050, which is a 77% rise from the year 2022 [1,2,3]. The development of chemotherapy began in the 1940s with the recognition that exposure to nitrogen mustard (used as toxic gases in battle) depleted bone marrow and lymph nodes. The ensuing decades led to the development of multiple chemotherapy drugs to treat both hematologic and solid tumors, commonly prescribed intravenously in multi-drug combinations for increased potency [4]. Debilitating side effects, such as severe nausea and vomiting, hair loss, cachexia, weakness/fatigue, and organ damage or dysfunction (causing even more deadly diseases, such as heart failure) [5,6], have contributed to the widespread fear of even the word “cancer”. A few short years ago, traditional chemotherapy treatments rarely extended life beyond a year in people with advanced cancer [7]. Discoveries in cancer biology have improved the understanding of cancer proliferation drivers, spurring the development of medications “targeting” these alterations [8]. Since the 2000s, the availability of such targeted agents has grown exponentially, extending the lives of people for years with an entirely new chronic disease, that of advanced, metastatic cancer [9,10,11]. Indeed, metastatic cancers that were once rapidly deadly are currently stabilized with targeted therapy, often for years with many now dying from unrelated causes, such as old age [12].

Immunotherapy is a form of cancer treatment that utilizes the body’s immune system to identify and fight cancer cells [13]. Unlike traditional therapies, such as chemotherapy and radiation as mentioned above, immunotherapy enhances the body’s immune system activity through specific targets, minimizing harm to healthy cells while inhibiting cellular targets across many malignancies [14,15,16]. While immunotherapy is viewed as a novel treatment, its origins date back to the 19th century, when Dr. William Bradley Coley, the “Father of Immunotherapy”, first harnessed the immune system for the treatment of bone cancer, in 1891 [17,18]. Dr. Coley injected bacterial toxins, known as “Coleys toxins” into patients’ cancer cells, stimulating an immune response and laying the early foundation of immunotherapy [17,18]. In the decades following this, many immunology discoveries have been made, such as the discovery of dendritic cells, natural killer cells, interferons, tumor specific antigens, and ICIs, allowing for further advancement of the critical concepts of immunotherapy [17]. These foundational discoveries and research efforts have led to the development of cancer-specific immunotherapy, which has transformed cancer treatment and survivorship. Current immunotherapy approaches include immune checkpoint inhibitors (ICIs) and chimeric antigen receptor T-cell therapy, which involves genetically modifying T-cells in the body to target specific cancer antigens [17,18]. Immune checkpoints are a part of the immune system, and they engage when proteins on the surface of T-cells bind to proteins on other cells, including tumour cells. These are referred to as “immune checkpoints”, as this process prevents T-cells from destroying the binding cell. ICIs block the checkpoint proteins from binding, allowing the T-cells to fight cancer cells [18]. ICIs are a newer and more promising type of immunotherapy; the first ICI (CTLA-4 checkpoint inhibitor) received FDA approval for the treatment of metastatic melanoma in 2011 [18].

Specific to ICIs, patients commonly experience long lasting anti-cancer effects alongside a much more tolerable toxicity profile compared to traditional chemotherapy drugs (whole-body deleterious effects, including nausea, vomiting, diarrhea, alopecia, pancytopenia, etc.). In contrast, ICIs work by reinvigorating the host immune system to fight tumour cells [19]. Despite better patient tolerance, ICIs can result in unique toxicities referred to as immune-related adverse events (irAEs) as a result of immune system dysregulation [20]. Drugs targeting CTLA-4, PD-1 and PD-L are some of the most common agents, prescribed in mono- or combination therapy. Patients may remain on these treatment protocols for their entire lives, changing treatment only when one regimen becomes ineffective in maintaining tumour “stasis”. With such success, the chronic nature of these treatments presents an entirely new challenge and patient population, specifically the ongoing monitoring of potential side effects, often for years. Nurses have central roles and responsibilities within the cancer care continuum, including prevention, screening, detection, and treatment. Their roles vary, often depending on the setting of practice (e.g., ambulatory care, acute care clinic, community or home healthcare), specialty (e.g., oncology, palliative care), healthcare systems setting (e.g., rural or urban regions), organizational policies, and patient population. The aim of this paper is to discuss the essential role of nursing in the short- and long-term monitoring of people receiving cancer immunotherapy, specifically ICIs. Therefore, this paper will not review the mechanisms of action but focus on the role of nurses in the assessment of side effects, identifying challenges patients face and the need for intervention, and providing education for self-management. This paper provides a unique Canadian perspective on oncology nursing, overviewing the Canadian landscape of oncology nurses’ roles when caring for patients receiving immunotherapy, specifically ICIs.

## 2. Patient Education

Nurses play a critical role in patient education and supporting health literacy, ensuring patients understand their treatment, potential symptoms and side effects. Immunotherapy is an evolving and highly complex form of cancer treatment, making patient education even more challenging. There may be a false belief that such agents are not as “strong” as traditional chemotherapy drugs, as fewer debilitating side effects may be experienced. Oncology nurses often take the lead on educating patients on the early signs and management of irAEs [21,22].

Education ensures patients are empowered with the ability to identify early signs of adverse events and seek appropriate intervention when necessary [23]. Education incorporating various formats (written, oral, and digital) that enhance ICI understanding are crucial in improving early detection and irAE management [23]. This is essential, as early recognition and management is key in limiting potential organ damage [20]. Wherever possible, nurses should assess and identify learning strategies best suited for their patients to provide effective education [24]. In some jurisdictions, group ICI teaching programs have been developed, aiming to efficiently deliver standardized education and facilitate group discussions amongst people embarking on similar treatment. Written information to complement the learnings is essential, as the stress associated with a new treatment protocol or new cancer diagnosis can hamper retention. Well-delivered education on irAEs has the potential to decrease patient stress and anxiety, as patient education in general oncology that includes side-effect management has shown to decrease overall patient anxiety [25]. An emergency contact card outlining severe irAEs and urgent contact numbers should be given to patients. This can be supplemented with an ICI-specific symptom guide that highlights which symptoms require immediate emergency department attention or those that can be managed by a phone call to the cancer centre. In addition, an emergency triage letter indicating a patient’s specific ICI and outlining potential irAEs can provide significant benefit in guiding community, urgent care, and emergency room providers who may be unfamiliar with ICI therapy. This letter should also include an urgent contact number for consulting a designated, on-call provider experienced in irAE management.

Patients starting cancer immunotherapy are often monitored closely when initiating treatment. Over time, follow-up care is often transitioned to nurses or general practitioners within their communities, placing greater responsibility on the patient. While uncommon, some patients may not immediately react to their therapy and instead experience irAEs in the later stages of their treatment, making them more complex and challenging for the patient. These challenges further highlight the need for patient education to allow the patient to understand their treatment, monitor their symptoms, and catch early signs of irAEs, preventing health consequences.

## 3. Symptom Assessment and Monitoring

Nurses play a pivotal role in monitoring patients for the early detection and management of these events, which greatly affect patient health outcomes. Nurses should conduct systematic assessments at each visit, aiming to recognize early signs of irAEs [26]. Early interventions may potentially prevent unnecessary treatment delays or discontinuations, thus promoting treatment continuity and preventing poor health outcomes.

**Figure 1:**
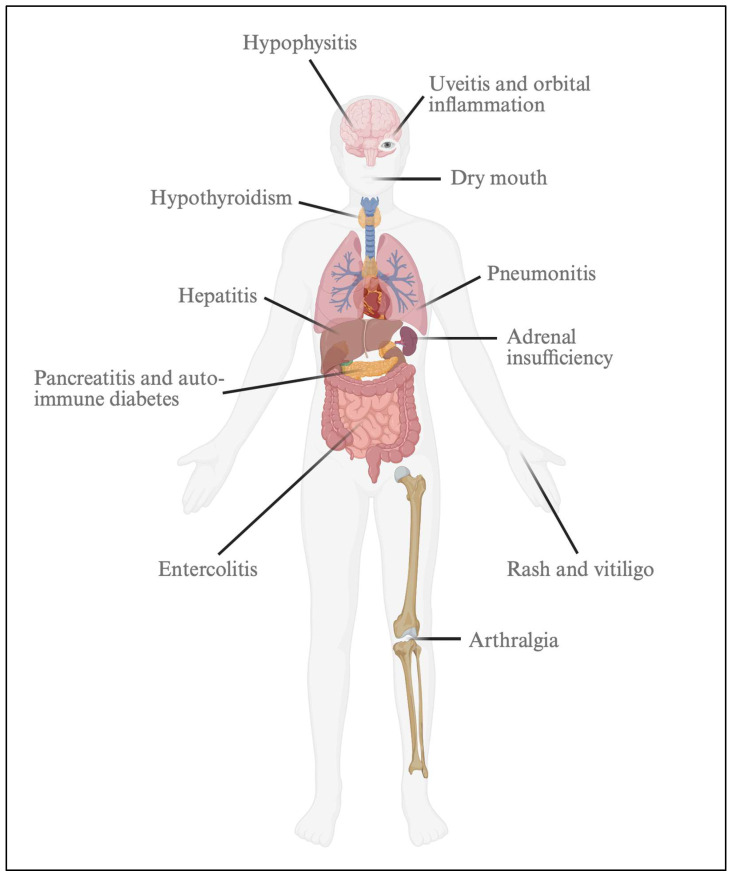
Immune-related adverse events with immune checkpoint blockade. Created in BioRender. Mirzadeh, P. (2025) https://BioRender.com/xdz0dvf.

A detailed history of the treatment cycle experience and trajectory of any symptoms should be effectively explored. The particular challenge associated with ICIs is that any organ system may be vulnerable to inflammatory events, thought to be related to ICI effects on T-cell activation and functioning (Figure 1) [27].

A key question to consider is “what do you find different from your last assessment?”, with the goal of avoiding issues potentially overlooked with typical checklists. In-person general surveys should always be performed whenever possible, aiming to observe unexpected or additional effort with breathing, speaking, or ambulation. Weight and complete vital signs are essential components of the assessment, aiming to identify changes from baseline functioning. Provocative testing with pulse oximetry during a walking test may be considered according to nursing expertise. Careful assessments and serial vital sign evaluations may detect early effects consistent with myocarditis or pneumonitis. Any new or changed medications should be reported. Ideally, provincial electronic medical records will allow for continuous and readily available data accessible to all potential health settings, as is the case in Alberta.

There are several innovative methods of symptom monitoring and management provided by nurses, such as nurse-led clinics (with nurse practitioners) and virtual clinics. One example, CareChart Oncology, is an oncology tele-triage line that operates on weekends, evenings, and holidays when clinics are typically closed [28]. Patients experiencing symptoms from the treatment can call, and this service will connect the patient with a specialized oncology nurse who triages the patient [28]. These nurses use evidence-based triage tools, such as COSTaRS, the pan-Canadian Oncology Symptom Triage and Remote Support practice guides that provide guidance on symptom management and support to enhance quality and consistency of care provided by nurses [29]. This service is beneficial and allows nurses to mostly manage calls through patient self-management strategies and relays the information to the patients’ hospitals for continuity of care [28]. Ontario Health shared that patients who utilized this service reported having a good experience and expressed high satisfaction [28]. Another example is weekly telephone calls from nurses to patients receiving immunotherapy, where nurses use a checklist to virtually assess toxicity [30]. These proactive nurse-led programs have also been shown to be both feasible and well accepted [30].

While there is growth in the number of nurse-led services in oncology where nurses provide specialized care for patients with cancer, including those undergoing immunotherapy, there is limited data on the usefulness of these clinics specific to cancer immunotherapy, specifically ICIs, and there is a growing trend of these models of care in ambulatory settings. Most of the existing literature explores the role of the nurse in both immunotherapy and traditional therapy, rather than focusing solely on immunotherapy. Further research is needed to explore the role of nurses in immunotherapy, as it differs from traditional treatment methods, potentially causing irAEs, ranging in severity and requiring close monitoring and management [31]. Examples of side effects and symptoms following cancer ICI and chemotherapy are shown in Table 1. Understanding the distinct responsibilities and challenges faced by nurses in symptom monitoring and management is essential for optimizing patient health outcomes and developing effective evidence-based nursing interventions for the early detection of adverse side effects.

Electronic patient-reported outcomes (ePROs) are a method of obtaining real-time reports of patient symptoms in a timely and continuous manner [32,33], and they are often used in cancer immunotherapy. Specific examples include the digital platform V-Care developed by Moradian and colleagues, which uses ePROs to create a novel follow-up pathway for cancer immunotherapy [34]. This digital platform allows for communication between the healthcare team and the patient, allowing for more personalized and comprehensive care [34]. While the use of ePROs has shown to improve patients’ quality of life [35,36] and positively affect patient and healthcare provider communication, enhancing education and self-management [37], the specific use of ePROs in the context of immunotherapy cancer treatment and its effects on patient health outcomes remain unclear and warrant further investigation.

## 4. Supportive Care

Oncology nurses provide ongoing supportive care for their patients undergoing cancer ICIs. As described, ICIs pose unique challenges in the form of irAEs, which can be severe or life threatening. In addition to educating patients and monitoring their symptoms, nurses provide additional psychosocial support, as well as coordination and navigation of multidisciplinary care teams. Receiving ICI therapy can be mentally tolling; therefore, nurses can reassure patients embarking on ICI therapy that although irAEs are common, when they are managed appropriately, overall survival and patient outcomes are often not inferior and, in some cases, may even be better [38]. Additionally, nurses actively listen to patients and offer emotional support, connect patients with support groups or psychosocial oncology, and in some cases, lead the support groups. Furthermore, in cancer care, patients are often responsible for managing their own care due to the care shift to the community setting. Nurses play a key role in supporting these patients experiencing irAEs by coordinating and facilitating referrals to multidisciplinary team members, including dieticians, physiotherapists, occupational therapists, social workers, and psychologists. For example, dieticians can help to support patients recovering from autoimmune colitis, while psychologists can assist in the management of the emotional distress associated with ICI rechallenge after the onset of irAEs. In these multidisciplinary settings, nurses can serve as a central point of contact and liaise between the various allied health professionals to improve continuity and quality of care. In addition, nurses can connect patients with cancer-focused community programs that support mental health, social, educational, exercise, or even recreational needs, such as those provided by the various local and national organizations across Canada.

Limited research is available on the specific role of nurses in providing supportive care to patients receiving ICI therapy, outside of education and symptom monitoring. Specific to nursing support, there is a variety of literature in other areas, such as intensive care, labor and delivery, and surgery; however, nursing support in ICI therapy is limited, likely due to the novelty of ICI therapy.

## 5. Conclusions

In summary, cancer immunotherapy is a newer form of cancer therapy that utilizes the body’s immune system to fight cancer cells. While immunotherapy is an effective treatment against cancer, there is also the possibility of irAEs. Oncology nurses have a central role in caring for patients receiving immunotherapy, including educating patients on detecting signs and symptoms of irAEs, assessing and actively monitoring patients, and providing support in the form of emotional support and coordinating care with the multidisciplinary care team. Several Canadian resources are linked below, for healthcare providers seeking additional information on caring for patients receiving cancer immunotherapy.

While specialized oncology nurses are well equipped to care for patients undergoing ICI therapy, further research is needed on interventions to improve ICI understanding and irAE recognition for nurses in general community, urgent care, emergency, and acute care settings, as cancer patients undergoing these treatments may end up in the care of any of these centers for a myriad of cancer-related or unrelated reasons. Given that ICIs are a newer form of immunotherapy and first received FDA approval in 2011, there is a gap in the literature in assessing care specific for patients receiving ICI therapy, in the new area of technology in healthcare. Nurses provide care through a variety of channels, including telephone triage lines and nurse-led clinics, and they provide care through a variety of means; however, there is limited literature assessing these supports specific to ICI therapy. Future works should fill these gaps by examining the barriers and facilitators of nursing care in ICI therapy.

Finally, while this paper offers insights into the perspectives and experiences of oncology nurses caring for patients receiving ICIs, nursing roles and practices may vary across different immunotherapies and across global healthcare settings.

List of Canadian Resources (current at time of publication)

- Cancer Care Ontario Immune Checkpoint Inhibitor Side Effect Toolkit;
- BC Cancer: Immunotherapy Checkpoint Inhibitors (guide for nurses):
  ■ BC Cancer Drug Index;
- Cancer Care Alberta (information for patients and families);
- Canadian Association of Nurses in Oncology (immuno-oncology essentials for oncology nurse);
- Canadian Cancer Society;
- Well Spring (patient support organization).

## Figures and Tables

**Table 1:** Examples of side effects and symptoms following cancer immune checkpoint inhibitors and chemotherapy.

Immune Checkpoint Inhibitors	Chemotherapy
Flu-like symptoms ○ Fever ○ Chills ○ Fatigue ○ Weakness ○ Headache Immune-system reactions ○ Infusion reactions ○ Pneumonitis ○ Hepatitis ○ Colitis ○ Rashes ○ Arthritis and myalgias ○ Ocular toxicity Hormonal changes ○ Thyroid dysfunction ○ Hypophysitis ○ Adrenal insufficiency	Myelosuppression Febrile Neutropenia Bleeding events Thrombosis Nausea and vomiting Mucositis Diarrhea Constipation Fatigue Infusion reactions Hair loss Cardiac toxicity Pulmonary toxicity Hepatotoxicity Nephrotoxicity Neuropathy Hand–foot syndrome Secondary malignancies

## Abbreviations

The following abbreviations are used in this manuscript:

ICI	Immune checkpoint inhibitors
irAEs	Immune related adverse events
ePROs	Electronic patient-reported outcomes
